# The Role of Plant Growth-Promoting Bacteria in Alleviating the Adverse Effects of Drought on Plants

**DOI:** 10.3390/biology10060520

**Published:** 2021-06-11

**Authors:** Khaled Abdelaal, Muneera AlKahtani, Kotb Attia, Yaser Hafez, Lóránt Király, András Künstler

**Affiliations:** 1 Excellence Center (EPCRS), Plant Pathology and Biotechnology Laboratory, Faculty of Agriculture, Kafrelsheikh University, Kafr Elsheikh 33516, Egypt; yasser.abdelgwad@agr.kfs.edu.eg; 2 Biology Department, College of Science, Princess Nourah Bint Abdulrahman University, Riyadh 11564, Saudi Arabia; mdfkahtani@gmail.com; 3 Center of Excellence in Biotechnology Research, King Saud University, Riyadh 11451, Saudi Arabia; kattia1.c@ksu.edu.sa; 4 Centre for Agricultural Research, Plant Protection Institute, ELKH, 15 Herman Ottó Str., H-1022 Budapest, Hungary; kiraly.lorant@atk.hu (L.K.); kunstler.andras@atk.hu (A.K.)

**Keywords:** plant growth-promoting bacteria, drought, antioxidant enzymes, chlorophylls, phenols

## Abstract

**Simple Summary:**

Among abiotic stress factors, drought is one of the most detrimental factors in arid and semiarid regions, causing a significant decrease in plant growth and yield in most species, including crops. Under drought conditions, morphological, physiological and biochemical characteristics such as plant height and enzymatic activities are negatively affected. This negative effect may be alleviated with the aid of plant growth-promoting bacteria. Application of plant growth-promoting bacteria such as *Paenibacillus*, *Azospirillum*, *Rhizobium*, *Bacillus*, *Azotobacter*, *Klebsiella*, *Pseudomonas* and *Serratia* can enhance hormonal balance, maintain nutrient status and improve plant growth characters as well as increase yield. This review discusses the pivotal role of plant growth-promoting bacteria in mitigating drought stress by improving plant growth characters and yield.

**Abstract:**

Plant growth-promoting bacteria play an essential role in enhancing the physical, chemical and biological characters of soils by facilitating nutrient uptake and water flow, especially under abiotic stress conditions, which are major constrains to agricultural development and production. Drought is one of the most harmful abiotic stress and perhaps the most severe problem facing agricultural sustainability, leading to a severe shortage in crop productivity. Drought affects plant growth by causing hormonal and membrane stability perturbations, nutrient imbalance and physiological disorders. Furthermore, drought causes a remarkable decrease in leaf numbers, relative water content, sugar yield, root yield, chlorophyll *a* and *b* and ascorbic acid concentrations. However, the concentrations of total phenolic compounds, electrolyte leakage, lipid peroxidation, amounts of proline, and reactive oxygen species are considerably increased because of drought stress. This negative impact of drought can be eliminated by using plant growth-promoting bacteria (PGPB). Under drought conditions, application of PGPB can improve plant growth by adjusting hormonal balance, maintaining nutrient status and producing plant growth regulators. This role of PGPB positively affects physiological and biochemical characteristics, resulting in increased leaf numbers, sugar yield, relative water content, amounts of photosynthetic pigments and ascorbic acid. Conversely, lipid peroxidation, electrolyte leakage and amounts of proline, total phenolic compounds and reactive oxygen species are decreased under drought in the presence of PGPB. The current review gives an overview on the impact of drought on plants and the pivotal role of PGPB in mitigating the negative effects of drought by enhancing antioxidant defense systems and increasing plant growth and yield to improve sustainable agriculture.

## 1. Introduction

Among abiotic stresses, water deficit or drought is considered as one of the most severe factors that hampers growth of various plants, particularly in arid and semiarid regions. The effects of drought occur on plants due to many factors, including lack of irrigation water, low rainfall, low and high air temperatures as well as salinity. Furthermore, drought symptoms may appear on the plant in spite of the presence of sufficient amounts of water in the soil which the plant cannot absorb. This phenomenon is called physiological drought. Drought stress causes a decrease in the quality of morphological, physiological and biochemical characteristics, and consequently, decreases crop growth and yield [1,2]. In many regions, annual precipitation has been reduced due to global warming, resulting in negative effects on plants [3] such as decreased relative water contents (RWC) and turgor loss [4,5,6]. One of the first reactions of a plant exposed to drought is the closure of stomata and a reduction in CO_2_ concentrations and photosynthesis, potentially leading to plant death under severe stress [7]. The decline in leaf development under drought also harmfully affects photosynthesis, since decreases in water contents are accompanied by a reduction in relative water contents [8]. In addition, respiration, ion uptake, carbohydrate and nutritional assimilation are negatively affected under water deficit stress [9,10]. Drought stress causes a significant decrease in stem length, leaf area and grain yield in faba bean plants [9]. RWC and concentrations of Chl. *a* and Chl. *b* significantly decreased in, e.g., canola plants under water deficit stress [11]. Furthermore, electrolyte leakage, lipid peroxidation and levels of ROS like superoxide (O_2_^•−^) and hydrogen peroxide (H_2_O_2_) were considerably elevated in maize plants exposed to a water deficit [12]. Under drought conditions, plants close their stomatal pores, decreasing the uptake and fixation of CO_2_, which causes profound changes in metabolism, mainly photosynthesis, and an extreme increase in the formation of reactive oxygen species (ROS) (i.e., enhanced oxidative stress) [13,14]. Plant cells contain efficient defense systems to scavenge ROS; these systems of enzymatic and nonenzymatic antioxidants play essential roles in protecting chloroplasts and mitochondria against the oxidative stress caused by various stress factors [15,16,17,18,19,20,21,22]. Furthermore, the negative effects of abiotic stresses—in particular, drought—can be successfully overcome in plants by plant growth-promoting bacteria (PGPB), which are natural habitants of the rhizosphere soil. The most well characterized plant growth-promoting (PGP) genera/species are *Rhizobium*, *Azospirillum*, *Bacillus*, *Azotobacter*, *Paenibacillus*, *Pseudomonas*, *Serratia*, and *Klebsiella*. Most of these bacteria have the capability to enhance growth characters and yield under natural conditions by nitrogen fixation, production of amino acids and phytohormones as well as improving nutrient availability in many plants exposed to biotic and abiotic stress [23,24]. Thus, PGPBs have the ability to convert unfertile soils to fertile soils and confer enhanced plant adaptation to various stresses such as diseases, drought, salinity, extreme temperatures and light [25,26,27] by production of gibberellins, indole acetic acid (IAA), cytokinins, 1-aminocyclopropane-1-carboxylate (ACC) deaminase, siderophores and several essential nutrients, especially phosphorus, zinc and manganese. Application of PGP microbes can ameliorate drought stress in many plants such as wheat [28], rice [29] and maize [30], primarily by increasing nutrient availability. Overall, PGPBs as biofertilizers provide a cheap and ecofriendly technique to improve plant growth and development under drought conditions, so it makes them an important and essential tool to facilitate sustainable agriculture. In this review, we focus on the role of PGPBs as an alternative system to (1) reduce the application of chemicals such as herbicides, pesticides and chemical fertilizers which cause damage to the soil and human health, affect water quality and result in environmental pollution, and (2) alleviate the negative effects (e.g., oxidative stress) of drought, one of the most harmful factors that hamper yield improvement and agricultural sustainability.

## 2. Morphological, Anatomical, Physiological and Biochemical Responses to Drought Conditions

### 2.1. Morphological and Anatomical Responses

In plants, one of the first morphological responses to drought is impaired seed germination and weakened seedlings [31]. On the other hand, increasing root length and the number of roots are important features for a plant to enhance the shoot system by improving water availability, as shown for, e.g., rice plants [32]. A well-established root system is part of a type of adaptation mechanism to drought called drought avoidance [33,34].

**Figure 1:**
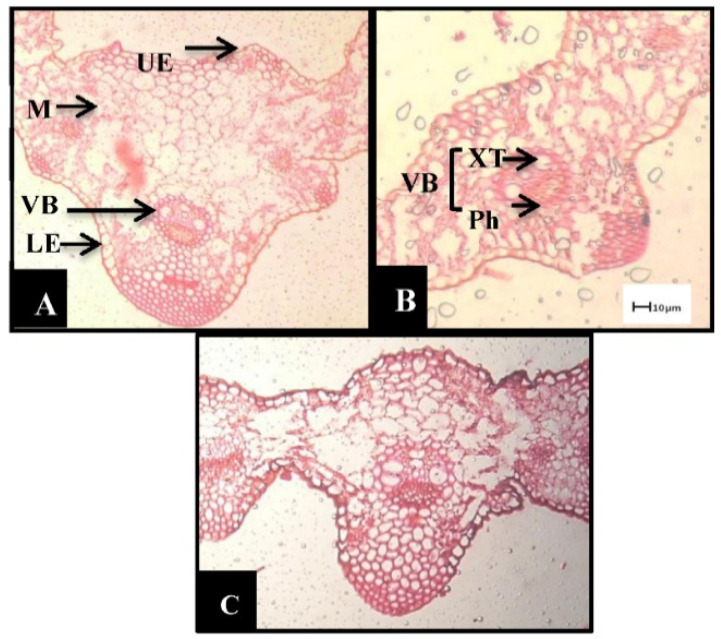
Effects of drought on barley leaves (transverse sections). (**A**) Control. (**B**) Plants irrigated once (D1), (**C**) plants irrigated twice (D2) (X 200). UE: upper epidermis, MT: mesophyll tissue, VB: vascular bundles, LE: lower epidermis, XT: xylem tissue, PhT: phloem tissue. (Hafez et al. [10]).

**Figure 2:**
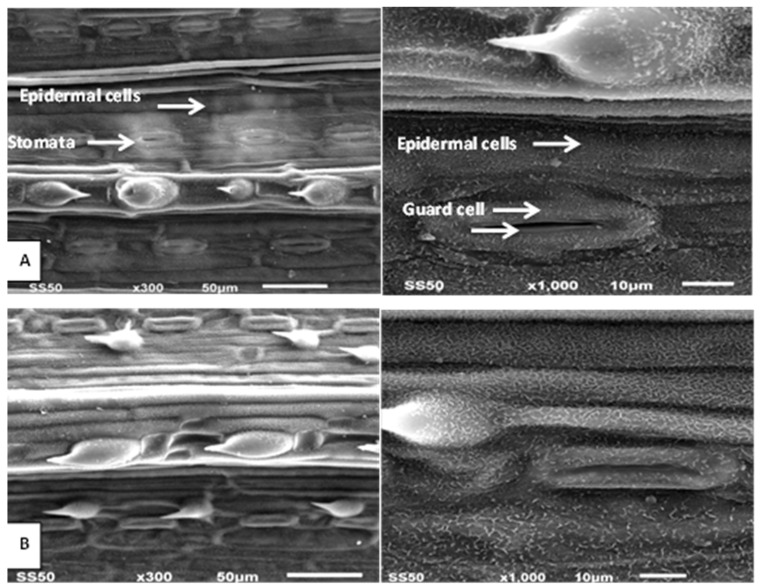
Effects of drought on barley leaves (Scanning Electron Microscope image). (**A**) Control. (**B**) plants irrigated once. Bar = 10 μm, Bar = 50 μm. (Abdelaal et al. [2]).

As a response to drought, the reduction in leaf size and numbers as well as numbers of stomata was also recorded in many plants [32,33,34,35]. A remarkable impairment in morphological characters such as leaf area and plant height was shown to occur under drought stress in faba bean plants [9], wheat [1], barley [2,10] and flax [8]. Plant growth and development are usually associated with plant cell elongation and division, which are negatively affected by drought, thus damaging cellular differentiation and plant growth as well as yield [2,5,9]. Similar results were observed in flax [36], sugar beet [37] and wheat [38]. The fact that leaf area is negatively affected under drought conditions could be due to the reduction in leaf numbers, size and longevity, depending also on temperature, leaf turgor pressure and assimilation rate [39,40]. The reduction in fresh and dry biomass is a common negative effect of drought [41] and observed in many plants [42,43]. Furthermore, flower numbers, plant height and shoot dry weight are also significantly reduced under drought stress [44]; consequently, the quality of yield components decreases [2,10,45,46]. The above-mentioned morphological responses are usually combined with anatomical changes in plants exposed to drought such as thickening of cell walls, increased cuticle formation on the leaf surface as well as improved development of vascular tissues [2,9,10,45]. Our previous results showed that drought stress resulted in anatomical alterations in the lower and upper epidermis, mesophyll tissue and vascular bundle diameter of leaves (Figure 1 and Figure 2). These effects may be due to a shortage of water supply from the soil, nutrient uptake reduction, and a reduced photosynthetic rate, consequently negatively affecting the anatomical characteristics of barley leaves [2,10]. Several studies have reported that during drought stress, plant hydraulic conductivity can change as a result of disruption of the water flow in the xylem vessels (embolism) or modifications in vessel size and function [47,48,49]. These anatomical changes may reduce water flow from the root to the shoot system [50] and consequently promote stomatal closure and limit transpiration [51].

### 2.2. Physiological and Biochemical Responses to Drought Stress

#### 2.2.1. Physiological Responses

##### Chlorophyll and Photosynthesis

**Figure 3:**
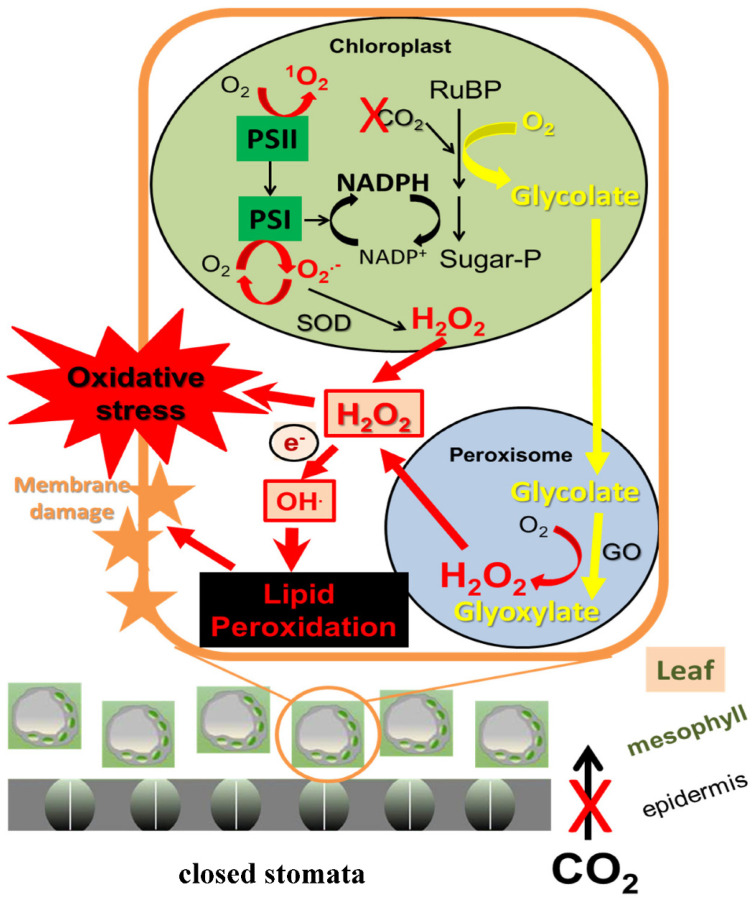
Drought stress may cause excessive accumulation of ROS due to perturbations in photosynthesis, resulting in oxidative stress within plant cells. Drought-induced closure of stomata restricts carbon dioxide (CO_2_) uptake, causing overaccumulation of reduced photosynthetic electron transport components (e.g., NADPH) in chloroplasts and increased oxygenation of ribulose-1,5-bisphosphate (RuBP). This accelerates the production of glycolate. In peroxisomes, glycolate is converted to glyoxylate and hydrogen peroxide (H_2_O_2_) by glycolate oxidase (GO), accounting for most of peroxisomal H_2_O_2_ production in green tissues of C3 plants. These processes also favor the chloroplastic accumulation of the ROS superoxide (O_2_^•−^), singlet oxygen (^1^O_2_) and H_2_O_2_ by the photosynthetic electron transport chain (PSI and PSII). H_2_O_2_ from chloroplasts and peroxisomes may be transferred to the cytoplasm, where massive oxidative stress and membrane lipid peroxidation may develop, especially due to the action of the H_2_O_2_-derived hydroxyl radical (OH^•^). Based on Noctor et al. [56] and Nadarajah [57].

One of the main effects of drought on physiological parameters is a decrease in Chl. *a* and *b* contents as well as of photosynthetic rate. The full development of the shoot system and sufficient stomatal opening are important factors for optimum photosynthesis; consequently, net photosynthesis is decreased due to a reduction in leaf numbers and an increase in leaf senescence during drought [52,53]. Plants expand the roots and create a branched root system to increase water uptake and overcome drought conditions [54,55]. Under drought stress, the stomatal pores are closed, resulting in a decreased carbon dioxide (CO_2_) uptake and causing a partial reduction in molecular oxygen, i.e., an increase in the production of ROS such as hydroxyl radicals (OH^•^), O_2_^•−^ and H_2_O_2_, which cause oxidative stress in, e.g., chloroplasts, peroxisomes and mitochondria under different stresses such as, e.g., drought [2,47,52,56,57,58], salinity [59,60,61,62,63,64] and phytopathogens [65,66,67,68]. Interestingly enough, the reduction in CO_2_ uptake led to a decrease in photosynthetic rate because of a decrease in the activities of enzymes that catalyze the dark reactions and the Calvin cycle pathway. Indeed, this negative effect on dark reactions may lead to an imbalance between the light and dark reactions as well as excessive ROS accumulation in plant organelles, especially chloroplasts and peroxisomes, where a restricted CO_2_ uptake during, e.g., drought, favors enhanced photorespiratory H_2_O_2_ production [46,57,58,69] (Figure 3). The ROS accumulation led to disturbances in thylakoid membrane structure, activities of enzymes and photosynthetic pigments [53,70]. Several reports indicated a notable decrease in Chl. concentrations that might be due to reduced Chl. biosynthesis as a response to drought stress. In fact, this reduction in Chl. synthesis could be due to the adverse impacts of drought on, e.g., ribulose 1,5 bisphosphate carboxylase oxygenase (RuBisCO), an essential enzyme of the Calvin cycle and 5-aminolevulinate dehydratase, which play a significant role in the pyrrole biosynthesis pathway necessary for Chl. production [2,9,71].

Moreover, one of the main effects of decreased photosynthetic rates is a reduction in the synthesis of Adenosine triphosphate (ATP), due to reductions in phosphorylation and regeneration of Nicotinamide adenine dinucleotide phosphate (NADP). Along with RuBisCO, there are some additional important enzymes in plant carbon metabolism, for example, phosphoenolpyruvate carboxylase, fructose-1,6-bisphosphatase, sucrose phosphate synthase, and pyruvate orthophosphate dikinase; the activity of these enzymes decreases along with relative water content during drought stress [56,72,73].

##### Phenolic Compounds

Under stress conditions, plants have a protective strategy to alleviate these adverse impacts; one of these strategies is inducing the biosynthesis of phenolic compounds [74]. These compounds, naturally found in plant cells, are produced in the cytoplasm and endoplasmic reticulum and play an important role in ROS scavenging under abnormal conditions [75]. This increase in the concentrations of phenolic compounds could be due to the accumulation of carbohydrates in cells exposed to water stress [76] and was observed during different pathways such as those related to malonic acid and shikimic acid. In fact, the accumulation of phenolic compounds was associated with the balance between carbohydrate sources and sinks. Moreover, the levels of flavonoids and phenolic compounds might be related to the morphological changes and metabolic alterations that protect plant cells from oxidative damage under water deficit [77]. Phenolic compounds were elevated under stress conditions to reduce the negative impact of stress by scavenging reactive oxygen species [78]. Drought led to an increase in vitamin C contents, total polyphenols and total flavonoid contents in *Amaranthus tricolor* plants [79]. In another study by Siracusa et al. [80] an increase in polyphenolic and flavonoid compounds was recorded in drought-stressed buckwheat. In addition, in drought-stressed sugar beet plants a remarkable increase in polyphenolic compounds was recorded [58].

##### Relative Water Content (RWC)

RWC is an important indicator of drought stress. RWC and transpiration rate are significantly reduced in plants exposed to drought. Drought-tolerant cultivars have an improved water use efficiency as compared to susceptible cultivars, this effect in drought-tolerant plants may be due to increased biomass accumulation and low evapotranspiration because of stomatal closing [81,82]. The reduction in RWC is one of the earliest responses to drought and is usually followed by a reduced leaf water potential and stomatal closing [56]. Furthermore, stomatal closing is associated with increased leaf temperatures which cause denaturation of proteins and damage membrane stability, photosynthesis, mineral nutrition, ion uptake and the synthesis of amino acids [83,84].

##### Mineral Elements

Mineral elements are very important factors for growth and differentiation in all stages of plant life. Drought stress affects the assimilation and uptake of minerals such as nitrogen, phosphorus, silicon, calcium and magnesium which may lead to reduced growth and development [85]. The fact that drought stress leads to stomatal closure and decreased photosynthetic rate eventually affects the export rate of sucrose from source to sink, consequently resulting in a reduced plant growth and suboptimal yields [86]. Numerous studies reported that drought harmfully affects nutrient availability in soils, reduces nutrient uptake and transport, decreases their concentrations in plant tissues and finally causes impaired plant growth [56,75]. In general, the plant plasma membrane plays an important role in mineral nutrition, and drought adversely affects membrane stability and mineral nutrition balance in plant cells, therefore, membrane stability is a pivotal factor in drought resistance [2,9,12,56,75].

##### Compatible Solutes

Compatible solutes (osmoprotectants or osmolytes) are natural compounds with low molecular weight, such as amino acids, sugars, glycine betaine or alcohols which are synthesized in the plant cytoplasm under both optimal and stressful conditions [87]. These compatible solutes play pivotal roles in osmotic adjustment by stabilizing proteins and cell structures as well as scavenging reactive oxygen species [88,89], consequently maintaining plant growth during various stresses. Biosynthesis of osmolytes such as polyols and betaines is a primary reaction of plant cells to tolerate drought stress. The accumulation of osmolytes will reduce water potential of cells and avoid detrimental ionic power, this maintains the water flow into the cell and regulates the turgor pressure thereby enhancing growth. Under drought stress, numerous amino acids, especially proline, accumulate in plant cells to deal with lack of water and protect cells from oxidative damage [90]. In addition, sugars such as sucrose, hexoses and raffinose are important osmolytes and contribute to membrane stability, thus sugars could protect plant cells against drought. Many oligosaccharides like raffinose and stachyose which play an essential role in drought tolerance were detected in seeds of several plant species [91]. In fact, plant sugars are now considered as ROS scavengers (antioxidants) since their reducing power contributes to the degradation of ROS like H_2_O_2_ [60]. For example, sugars like mannitol can protect chloroplasts from oxidative damage by inducing expression of abiotic stress-related genes that encode superoxide dismutase (SOD), heat shock proteins (HSP) and glutathione-S-transferases (GST). Another sugar, trehalose has been shown to regulate abscisic acid (ABA) metabolism and protect photosystem II (PSII) against excessive oxidation during various abiotic stresses in plants [60]. Glycine betaine (*N*,*N*,*N*-trimethylglycine) is a different type of important compatible solute synthesized via a two-step oxidation of choline and playing a pivotal role in stress tolerance by stabilizing macromolecules via maintaining intermolecular water balance [92]. Glycine betaine accumulation led to improved osmotic adjustment in transgenic plants, and accumulation of this compound depends significantly on the available choline in chloroplasts [93]. The effect of glycine betaine in decreasing malondialdehyde contents was observed to protect the plasma membrane. Additionally, application of choline may promote glycine betaine accumulation in plants because choline is the first compound in the glycine betaine biosynthesis pathway [94].

Proline is one of the most important amino acids, biosynthesized in plant cells as a response to various conditions, particularly drought stress, to regulate various processes and tolerate stress [2,62,63,92]. Proline formation in plants can occur via two biosynthetic pathways; the glutamate dependent pathway and the ornithine-dependent pathway. Many plants accumulate proline under unfavorable conditions, where proline concentrations in stress tolerant plants are higher than that in sensitive plants. In fact, plant mutants defective in proline production are more sensitive to drought [95]. Proline, as an osmolyte, stabilizes various components like proteins and membranes as well as scavenges reactive oxygen species (ROS) [2,14,17,19,47,92,93]. Together with sugars, prolines protect both plant photosystems (I and II) against oxidation during drought [60]. Szabados and Savoure [94] stated that proline plays a vital role in regulating mitochondrial functions, cell death and activation-specific gene expression that help plants to recover from stress. In general, drought stress affects turgidity and osmotic balance in plant cells, therefore, osmotic adjustment plays an effective role in plant life during drought with production of various compatible solutes (osmolytes) to alleviate drought stress-induced negative effects.

##### Phytohormones

It is well known that phytohormones play significant roles in regulating numerous processes in plant cells, particularly the interactions of plants with various stresses. These hormones include abscisic acid (ABA), jasmonic acid (JA), salicylic acid (SA) and melatonin. Abscisic acid (ABA) is an important hormone in the plant response to drought [95,96], it is synthesized in plant roots and chloroplasts. Under drought stress, ABA cannot move through the plasma membrane but may be transported into the guard cells of stomata and induce stomatal closure. This increase in ABA concentrations in guard cells may cause a decrease in water loss under drought conditions. Additionally, stomatal closure leads to reduced CO_2_ uptake, consequently, a decrease in the photosynthetic rate [45,59,78,97]. Furthermore, accumulation of ABA leads to a decreased accumulation of ethylene, cytokinin and gibberellin in drought stressed plants, while in fact cytokinins may help in delaying senescence [8,11]. ABA regulates osmotic balance and induces resistance to stresses via activating antioxidant genes such as catalase (CAT), superoxide dismutase (SOD) and peroxidase (POX) through ROS-induction, e.g., by increasing levels/activities of NADPH oxidase [98]. The pivotal role of ABA during drought was studied in several plant species like, e.g., rice (*Oryza sativa*) [85] and oilseed rape (*Brassica napus*) [95]. Similarly, drought stress causes an increase in the accumulation of another group of plant stress hormones, brassinosteroids that ultimately results in an increased water uptake, improved membrane stability and reduced ion leakage during drought conditions [99,100].

It has long been recognized that jasmonic acid (JA) is an important plant hormone under stress conditions, especially during drought stress tolerance, due to its role in regulating stomatal aperture. Furthermore, JA accumulation under drought is associated with an additional mechanism of ABA signaling which counteracts drought effects by decreasing transpiration rate [101]. Numerous studies revealed that salicylic acid (SA) contributes to the protection of photosynthetic mechanisms during various stresses like drought and salinity in several plants [2,9,38,47,64]. Similar results were also recorded in *Brassica rapa* [102] and *Triticum aestivum* [103] under drought conditions. In addition, treatments with SA (0.5 mM) significantly improved the growth of wheat seedlings by improving root length, plant biomass, decreasing lipid peroxidation and increasing AsA and GSH contents under drought conditions [104].

Melatonin (MEL) is an indoleamine (*N*-acetyl-5-methoxytryptamine) that was isolated from the bovine pineal gland in 1958 by Lerner et al. [105]. It is a strong antioxidant that occurs naturally and scavenges both reactive nitrogen species (RNS) and ROS in animal and plant tissues [106,107]. Melatonin pretreatment led to improved water status in plants, a decreased electrolyte leakage and increased photosynthetic rate. In addition, under stress melatonin may enhance the activities of antioxidant enzymes and scavenge H_2_O_2_ [108]. Furthermore, application of melatonin significantly decreased Chl. degradation and reduced the activation of *senescence-associated gene 12* (*SAG12*) under drought conditions [109]. Generally, it has been suggested that melatonin is a plant growth regulator, its action is similar to the hormone IAA and it can regulate the actions of other growth regulators. Melatonin may also protect plant organelles against reactive oxygen species and enhance membrane stability under harmful environmental effects due of its antioxidant properties [110].

#### 2.2.2. Biochemical Responses to Drought Stress

##### Oxidative Damage by Reactive Oxygen Species (ROS) Generation

Drought stress negatively affects plant cells and causes diverse biochemical changes, such as cellular membrane disorders, osmolyte production, ROS accumulation and increased activities of antioxidant enzymes [2,8,9,10,12,48]. OH^•^, O_2_^1^, H_2_O_2_ and O_2_^•−^ are the most well-known reactive oxygen species which accumulate as products of a partial reduction of atmospheric oxygen within the mitochondrial and chloroplast electron transport chains. During photosynthesis, the transfer of excitation energy from Chl. or univalent oxygen reduction at photosystem I in the Mehler reaction may produce singlet oxygen [111]. ROS can attack macromolecules causing harmful effects to lipids, nucleic acids and proteins resulting in cell death. It is well established that ROS play dual roles in plant life, they are important signaling agents under stress conditions at low concentrations but they act as toxic by-products and cause oxidative damage in, e.g., chloroplasts and mitochondria at higher concentrations [112]. Moreover, excessive accumulation of ROS damages photosystem II and obstructs D1 protein synthesis. ROS scavenging mechanisms can be orchestrated by antioxidants (enzymatic or nonenzymatic mechanisms) [113,114]. Enhanced enzymatic antioxidative mechanisms were observed with overproduction of some enzymes, for example CAT, POX, SOD and APX, leading to improved oxidative stress tolerance during, e.g., drought. However, nonenzymatic antioxidant mechanisms governed by α-tocopherol (vitamin E), flavonoids, ascorbate (vitamin C) and glutathione also have the capability to recover the plant after stress exposure, since these mechanisms aid in scavenging (detoxifying) ROS under stress conditions.

##### Oxidative Damage by Lipid Peroxidation (MDA Accumulation) and Electrolyte Leakage (EL)

Malondialdehyde (MDA) and electrolyte leakage (EL) are important signals of stress; these parameters considerably increase under various stresses such as salinity [60,61,62,63,64] and drought [8,9,12,58], resulting in deleterious effects on plasma membrane stability and selective permeability [2,37,46]. Furthermore, the increase in MDA could be due to the oxidative damage to chloroplasts and mitochondria, indicating an increased rate of lipid peroxidation. Accumulation of the highly reactive ROS, OH^•^, is the main initiator of stress-associated lipid peroxidation, resulting in severe injury of cell and organelle membranes that may lead to cell death [101]. Under water deficit stress, Hafez et al. [10] observed remarkable increases in MDA and EL in water-deprived barley plants, and this result may be due to the injury of membranes and desiccation of the cytoplasm. Additionally, Abdelaal et al. [46] found that MDA and EL levels significantly augmented as a response to drought in barley. Similar results were also recorded in several other plant species exposed to drought conditions [1,9,12]. In our recent study, drought stress led to significant increases in MDA and EL in sugar beet plants [58].

##### Antioxidant Enzyme Activities

Plant cells can cope with oxidative damage through antioxidant enzyme defense systems and nonenzymatic components, which scavenge high levels of ROS in different organelles [60]. Antioxidant enzymes have a critical importance in the scavenging of ROS and resistance to lipid peroxidation. The main enzymatic antioxidants are SOD, CAT, glutathione reductase (GR), ascorbate peroxidase (APX), dehydroascorbate reductase (DHAR) and monodehydroascorbate reductase (MDHAR). These enzymatic antioxidants can improve the physiological state of plant tissues by scavenging ROS or by inducing activities of other antioxidants to minimize stress-induced oxidative damage. The first enzyme involved in antioxidant mechanisms is SOD, which converts superoxide (O_2_^•−^) to H_2_O_2_, a less toxic ROS, and consequently, decreases damage to DNA and proteins [115]. According to the metal present in the prosthetic group, SODs are classified as Cu/Zn, Mn or Fe-containing enzymes (Cu/Zn-SOD (CSD), Mn-SOD (MSD) and Fe-SOD (FSD)). The results of SOD gene expression studies in stress-exposed wheat [116] and *Salvia miltiorrhiza* [117] show that SOD genes have different functions in mitigating stress effects in plants. The qRT-PCR results suggested that drought and salinity stress can improve or inhibit the expression of *HvSOD*s, which indicated that HvSODs have different mechanisms and differentially regulate the expression of downstream genes [118]. The expression of *HvCSD1* and *HvFSD1* decreased significantly under drought and salinity stress. On the other hand, the *HvCSD4* gene was significantly induced under drought stress and its expression increased nearly 70 times as compared to control plants. Wang et al. [119] reported that MSD is expressed in the peroxisomes and mitochondria, and CSD is mostly found in the chloroplasts, mitochondria and cytosol; however, FSD is mainly expressed in the peroxisomes, mitochondria and chloroplasts.

CAT (EC 1.11.1.6) is one of the most powerful antioxidant enzymes; it contributes to the degradation of H_2_O_2_ in a reaction where peroxide acts as hydrogen donor and acceptor [120]. Catalase plays a vital role together with SOD in removing O_2_^•−^ and in H_2_O_2_ degradation, its activity leading to a decrease in production of the highly reactive OH^•^. Peroxidase (POX) (EC 1.11.1.7) also contributes to H_2_O_2_ degradation by converting H_2_O_2_ to H_2_O. These antioxidant enzymes were shown to be involved in counteracting oxidative damage in wheat and barley during drought stress [1,9,12,58,121], while the decrease in antioxidant enzyme capacity is related to a reduced yield potential [122]. Peroxidases (POX) are important antioxidant enzymes involved in ROS scavenging in chloroplasts, mitochondria, peroxisomes and the nucleus. In fact, the increase in POX activities is associated with excessive formation of ROS during photorespiration and photosynthesis in peroxisomes and chloroplasts, respectively. Under drought conditions, POX activity significantly increases to mitigate the adverse effects on morphological and physiological characters in several plants [1,2,46,56].

##### Nonenzymatic Antioxidants

Nonenzymatic antioxidant compounds are low molecular weight molecules that effectively aid in increasing plant stress tolerance in response to adverse environmental factors [123]. Ascorbate (vitamin C), α-tocopherol (vitamin E), carotenoid and flavonoids are important examples of nonenzymatic antioxidant compounds that have the capability to enhance plant physiological status to effectively tolerate stress. Ascorbate can reduce OH^•^, H_2_O_2_ and O_2_^•−^ and may function as a substrate of antioxidant enzymes. It also participates in α-tocopherol production, zeaxanthin synthesis in the xanthophyll cycle and is a main component of the ascorbate–glutathione cycle [124]. In drought-stressed plants, ascorbate, along with glutathione, can scavenge H_2_O_2_ produced by photorespiration in peroxisomes [58]. Application of ascorbate in seed priming led to improved drought tolerance in wheat plants, which showed an increase in Chl. content, leaf area and dry weight under drought stress and these increases were associated with augmented proline levels [125]. Additionally, seed treatments with ascorbate led to improved salt tolerance in rice superior to that provided by other hormones such as kinetin and SA [126]. Glutathione is one of the major antioxidant components in plant tissues; it can play pivotal roles in ROS scavenging and also participates in the regulation of endogenous compounds in plant cells [57] such as, e.g., ABA signaling regulation. In Arabidopsis, treatment with glutathione causes an increase in ABA accumulation [127]; furthermore, treatments with glutathione enhanced drought tolerance and antioxidant responses under stress [128]. The ascorbate–glutathione cycle includes both nonenzymatic (ascorbate, glutathione, NADPH and H_2_O_2_) and enzymatic components (ascorbate peroxidase and glutathione reductase). Glutathione is a main component of the ascorbate–glutathione cycle, and its application can decrease the accumulation of OH^•^, H_2_O_2_ and O_2_^•−^ either directly or with glutathione peroxidase in a catalytic reaction [129].

## 3. Plant Growth-Promoting Bacteria (PGPB): Identification, Classification and Mechanisms of Action on Plants

PGPBs are members of the microbial communities related to plants growing under various conditions. These microorganisms use organic molecules from the rhizosphere such as sugars glutamine, betaine and trehalose to improve their growth. PGPBs include two groups: intracellular (iPGPB) and extracellular (ePGPB) [88]. ePGPB are the bacteria which improve the growth characters and colonize the root surface area or the intercellular space of the cortex. On the other hand, iPGPB are the bacteria which also stimulate plant growth but live specifically on the inside of the root surface/rhizoplant root cell nodular structure [86]. In 1926, endophytic growth was first characterized as an especially advanced step of bacterial lifestyle [130]. Later, several endophytes were isolated from surface-disinfected plant tissues [131]. ePGPB belong to the genera *Bacillus*, *Serratia*, *Azotobacter*, *Azospirillum*, *Micrococcus*, *Arthrobacter*, *Erwinia*, and *Pseudomonas*, while some iPGPB belong to *Rhizobia* such as *Allorhizobium*, *Mesorhizobium* and *Bradyrhizobium* as well as *Frankia* [132,133]. Endophyte bacteria are symbionts residing within the plant tissues for the majority of their life cycle without any detrimental impact on the host plant [134] and can be isolated from either the surface or inner plant tissues [135]. PGPB can promote plant growth by direct or indirect mechanisms. A direct promotion of plant growth is accomplished by increasing the nutrient availability such as that of nitrogen, phosphorous and iron as well as enhanced production of phytohormones [136]. An indirect mechanism is achieved by exerting antagonistic effects to tolerate numerous phytopathogens [137]. The direct strategy of PGPB includes nitrogen fixation, phosphate solubilization, and production of phytohormones and siderophores, which induce plant metabolism resulting in plant growth improvement [138], while the indirect strategy includes the increased activity of defense-related enzymes such as chitinase and β-1,3-glucanase, a reduction in endogenous stress-related ethylene (ET) and quenching the quorum sensing of phytopathogens [139,140]. Generally, the mechanisms of plant growth promotion may occur by solubilization of phosphorus, potassium and nitrogen uptake [141], and the regulation of phytohormones such as Indole-3-acetic acid (IAA) and gibberellic acid (GA_3_), zeatin, abscisic acid and ethylene, which maintain the root system and consequently, increase water uptake and nutrient availability. Several reports showed that PGPB may produce specific enzymes such as proteases and chitinases, which damage the cell walls of phytopathogens [142]. In addition, the production of antibiotics such as phloroglucinols, phenazines, pyoluteorin, pyrrolnitrin, and hydrogen cyanide (HCN), siderophores as well as bacteriocins was reported to help not only in inhibiting the development of phytopathogens [143], but also to confer enhanced plant tolerance to different stresses [144].

### 3.1. The Role of Plant Growth-Promoting Bacteria as Biofertilizers

A particular group of microorganisms that stimulate plant growth are called biofertilizers, which are phosphate-solubilizing and nitrogen-fixing microorganisms that can be applied to seeds, soil, or compost areas to increase the availability of nutrients by improving several microbial processes [124]. PGPB play a pivotal role in nutrient management in soils, which improves soil productivity and sustainability; furthermore, they are ecologically clean sources of nutrients, which can, at least partially, replace chemical fertilizers. Applications of PGPB significantly improve plant growth and production according to their role as biofertilizers [126]. The role of PGPB as biofertilizers was reported in several plants, including sugar beet [25,144,145] and sorghum [146]. Biofertilizers led to improved morphological, physiological and yield characteristics of sugar beet plants. The main role of PGPB as biofertilizers can be explained through phosphate and potassium solubilization and nitrogen fixation. Nitrogen fixation occurs by symbiotic or non-symbiotic microbes in association with plants [147]; for example, *Rhizobium* spp. [148], *Azoarcus* sp. [149] and *Serratia marcescens* [150]. Application of bacteria may enhance N_2_ fixation mediated by the *nif* gene along with other structural genes; they can activate plant growth, yield and improve disease management and maintain the nitrogen levels in the soil [151] and improve soil characters. Phosphorus is an essential element, and plays a key role in the growth and development including processes such as cell division and photosynthesis. The significant role of PGPB as biofertilizers was confirmed with *Rhizobium* (phosphate solubilization) and *Pseudomonas* and *Bacillus* spp. (phosphorus (P)-solubilizing bacteria) in plants. This role led to improved soil features and plant growth characters through organic acids which are secreted from these bacteria to facilitate a release of the bound forms of phosphates from calcareous soils by decreasing the pH in the rhizosphere. PGPB are environmentally friendly biofertilizers which not only increase and solubilize free phosphate and increase the fixation of biological nitrogen but also increase Fe and Zn availability in the rhizosphere as well as decrease the necessity of application of chemical fertilizers [152]. The phosphate solubilization process depends on C-P lyase, nonspecific phosphatases, and phytases, enzymes that act through the formation of organic acids such as oxalate, acetate, succinate, glycolate and citrate [153]. The phosphorus solubilizing bacteria could be used to mitigate the harmful effects of abiotic stress in plants such as high/low temperatures, drought and salinity. Moreover, PGPB can solubilize the insoluble potassium from rock and silicate. Well-known members of potassium-solubilizing PGPB are *Paenibacillus* spp., *Ferrooxidans* spp., *Bacillus mucilaginosus* and *Pseudomonas* sp. [154]. Potassium is one of the major macronutrients in plant life; therefore, a decrease in potassium content leads to a reduced plant growth and suboptimal crop yields [154].

One of the important compounds associated with the role of PGPB as biofertilizers is siderophores. Siderophores are organic compounds of low molecular weight that are produced by these microbes under Fe-stressed conditions and are capable of chelating Fe. These iron-chelates are recognized by specific receptor proteins (IROMPs) and transported into the cell by their respective permeases [155,156], consequently enhancing rhizospheric iron concentrations and increasing Fe bioavailability in the soil. The effect on nutrient uptake is due to the solubilizing power of PGPB in the soil. Accordingly, there are different types of PGPB, such as P-solubilizing PGPB, K-solubilizing PGPB, etc. [157]. Besides functioning as biofertilizers, PGPBs can also confer tolerance of plants to various stresses [158]. In general, the application PGPB as biofertilizers is considered as an alternative method to chemical fertilizers, which have caused significant damage to plants, the environment and human populations. PGPB can be used as a seed treatment, seedling dipping and soil application to obtain a higher plant yield and improve soil health with sufficient nutrients and valuable microflora, thus maintaining agroecosystem sustainability. This can be achieved through substitution of 20–40% of chemical phosphorus and nitrogen, improvement of plant growth, restoring soil fertility and mitigating drought stress effects. PGPBs can be used as a combined application, but cannot be mixed with fungicides and insecticides.

### 3.2. The Role of Plant Growth-Promoting Bacteria in Drought Stress Tolerance

Several studies have been conducted to examine the effectiveness of microbial inoculations for enhancing plant growth under drought stress. It is well-known that PGPBs are effective for improving growth of numerous plants such as legumes, cereals and vegetables under stressful conditions [159]. PGPBs may be applied as a strategy to reduce the damaging impacts of environmental stresses on plant growth and productivity by improving nutrient uptake, and consequently, increasing environmental stress tolerance [160]. Several studies have proved the helpful role of rhizobacteria in alleviating the negative impact of various stresses on crop growth [161]. Among various stresses, drought is one of the most harmful for plant production in arid and semiarid regions [162]. PGPB are effective and helpful for improving plant growth under drought stress [163], at least in part due to the fact that rhizobacteria can produce exopolysaccharides such as alginate and cellulose, which have been shown to help in improving drought resistance [164]. Thus, exopolysaccharides may play a significant role under drought conditions in mitigating stress effects, both in plants and in microbial populations.

Exopolysaccharides are responsible for the establishment of the attachment zone between bacteria and root systems, soil particles as well as between different bacteria. Some PGPB produce EPS, which may act as a barrier around the roots and improve plant growth under salinity stress [165]. Application with *Enterobacter* sp. MN17 and *Bacillus sp*. MN54 such as seed treatment of quinoa (*Chenopodium quinoa*) seeds led to an improved plant growth under salinity conditions (400 mM NaCl) [166]. Additionally, it has been reported that *Marinobacter lypoliticus* SM19 and *B. subtilis* ssp. *inaquosorum* decreased salinity and drought stress effects in wheat [167]. Furthermore, PGPB secrete lipo-chitooligosaccharides; these molecules are produced by rhizobia and induced by flavonoids which exist in root exudates. In addition, inoculation of soybean plants with *Bradyrhizobium japonicum* 532C led to improved growth under salinity stress (36 mM and 61 mM NaCl) [168]. Trehalose is a non-reducing disaccharide and highly stable molecule found in bacteria, fungi, plants and insects; it plays a significant role in improving plant tolerance to numerous abiotic stresses, mainly drought and salinity. Trehalose is a highly stable molecule and is resistant to high temperatures and acidity; it can decrease the damage caused by salinity and drought by preventing the protein aggregation and degradation that occurs under many stresses [169].

The growth promotion under drought stress may be also due to the fact that certain PGPB express ACC-deaminase, an enzyme which enhances the absorption of major nutrients such as N, P and K, consequently promoting plant growth under environmental stresses [170]. Application of the PGPBs *Herbaspirillum seropedicae* and *Azospirillum brasilense* improved drought tolerance in maize [161]; this positive effect may be due to the increase in water use efficiency and enhanced activity of antioxidant enzymes under drought stress. Furthermore, PGPBs cause improvements in growth characters due to producing plant growth hormones such as GA, IAA and cytokinins, resulting in increasing nitrogen fixation and improving nutrient absorption [171]. Additionally, PGPB play vital roles in mitigating drought stress tolerance due to abscisic acid (ABA) accumulation. Likewise, PGPBs accumulate antioxidants and osmoprotectants which can improve root growth as a response to stress [172,173,174]. Application of *Azospirillum* species led to improved growth of roots and increased lateral root formation under drought due to the production of indole acetic acid [164]. Inoculation of *Lavandula dentate* with *Bacillus thuringiensis* under drought stress led to increased nutrient uptake and enhanced metabolic activity in the plants [175]. Additionally, *Arabidopsis* and grapevine plants were adapted to drought stress due to inoculation with *Pseudomonas* and *Acinetobacter* species [176]. Foliar application with *Bacillus* causes an increase in ABA, induces stomatal conductance and increases water content in *Platycladus orientalis* under drought [177,178]. In another study, inoculation of soybean plants with *Pseudomonas* was shown to increase fresh weight and stem height under water deficit stress, with parallel increases in concentrations of Chl., SA and ABA, as compared to control plants [179]. Furthermore, application of *H. seropedicae* and *A. brasilense* in wheat cultivars led to maintenance of relative water contents, improved membrane stability as well as increased drought tolerance [180] associated with multiple mechanisms, including activation of antioxidant systems and osmolyte accumulation [181], as well as ACC-deaminase and hormonal activity [182]. It has also been stated that the useful effects of PGPB on plants such as barley (i.e., increased stress tolerance) are mediated by the accumulation of proline and several compatible solutes (osmolytes) [183].

Under drought stress, PGPBs can induce ACC deaminase activity directly or indirectly which enhances plant growth [184]. This mechanism depends on the consumption of ACC by PGPBs before its oxidation by ACC oxidases produced in plants. Therefore, PGPBs might be an excellent source of growth promoters and stress tolerance, since they are capable of reducing ethylene concentrations [185]. In wheat, application of PGPBs led to the improvement of plant growth by enhancing ACC deaminase activity and thereby regulating ethylene levels [186].

Inoculation of maize seeds with drought-tolerant, ACC deaminase-containing PGPBs significantly decreased the negative effects of drought stress and increased nutrient and water uptake from soils, consequently improving plant growth [187].

In another study, Bhattacharyya and Jha [188] reported that application of *Pseudomonas* sp. 4MKS8 led to improved agronomic characteristics of maize plants including root elongation. Additionally, application of *E. cloacae* 2WC2 helped plants to keep their water content and improve the root system in inoculated plants under water stress [189]. Drought stress led to enhanced electrolyte leakage in maize genotype TP 30 and caused an increased catalase activity as well as membrane damage, which could be due to oxidative stress. In this regard, application of *Bacillus* spp. led to decreased membrane damage, electrolyte leakage and increased membrane stability due to enhanced antioxidant enzyme activities in the drought-exposed maize plants [190].

Under salinity and drought stress, application of plant growth-promoting bacteria can deteriorate the harmful effects of stresses by production of cytokinin, ACC deaminase, trehalose, abscisic acid, organic compounds, and exopolysaccharides [191]. Different plant growth-promoting bacteria have been identified to improve the growth and yield of plants under drought stress (Table 1).

## 4. Conclusions and Future Perspectives

Widespread application of fertilizers and various chemicals to improve plant growth, soil fertility and crop yields has damaging impacts on agricultural soils, water resources, beneficial organisms, human health and the ecosystem. On the other hand, PGPBs, which include numerous bacterial species, can be successfully applied to improve growth characters and yield under various conditions by suppressing the negative effects of abiotic factors (e.g., heavy metals and drought) that eventually culminate in the excessive production of ROS causing oxidative damage. The beneficial influence of PGPBs on plants has been observed during numerous physiological processes such as phosphorus solubilization, nitrogen fixation, nutrient availability, production of vitamins, phytohormones and growth regulators. Consequently, PGPBs increase plant growth and yield to cope with drought stress and human needs due to increased population growth. Therefore, PGPBs as an alternative method to fertilizers and chemical substances may play a significant role in improving soil characters, yield production and sustainable agriculture under drought stress. Additional research must be conducted on the use of bacteria as biofertilizers, as plant growth regulators under stress conditions, and to identify the most powerful bacterial strains, as well as to define optimal treatment methods (seed treatment, seedling treatment, foliar application or addition to soil), in order to increase crop productivity, save the environment and improve agricultural sustainability.

## Figures and Tables

**Table 1:** Role of different plant growth-promoting bacteria (PGPB) in induction of plant drought tolerance mechanisms.

Plant Growth-Promoting Bacteria	PGPB Mechanisms Contributing to Improved Plant Drought Tolerance	Mechanisms of Plant Drought Tolerance	References
*Azospirillum* spp.	increase the accumulation of glutamate and glycine betaine which can act as osmoprotectants	increase drought resistance via accumulation of osmoprotectants improve growth characters	García et al. [30]
*Pseudomonas* spp.	up-regulate the proline biosynthesis pathway produce exopolysaccharides	improve water potential increase plant biomass	Sandhya et al. [159]
*Azosperillum brasilense* SP-7	prevents water loss by stomatal closure improves cell membrane structure	increase carbon, nitrogen, and chlorophyll levels decrease abscisic acid and ethylene levels increase relative water content	Curá et al. [161]
*Pseudomonas chlororaphis* O6	produces exopolysaccharides (EPS) produces phytohormones and 1-aminocyclopropane-1-carboxylate (ACC) deaminase	increase the activity of antioxidants and volatile compounds, inducing accumulation of osmolytes	Vurukonda et al. [175]
*Bacillus subtilis*	improves relative water content produces plant growth regulators, especially cytokinin	improve water potential increase sugars, amino acids and organic acids	Liu et al. [178]
*Azospirillum brasilense Herbaspirillum seropedicae*	improve integrity of cells and relative water content increase the levels of phytohormones induce defense-related proteins and enzymes	induce the levels of phytohormones in treated plants increase defense-related proteins improve activities of enzymes, antioxidants and epoxypolysaccharides	Furlan et al. [181]
*Bacillus subtilis* (LDR2)	reduces ABA/ACC contents enhances IAA contents maintains the optimal transpiration rate	improve photosynthetic efficiency regulate the expression of a regulatory component (CTR1) of the ethylene signaling pathway.	Barnawal et al. [182]
*Pseudomonas* sp. RJ15 *Bacillus subtilis* RJ46	regulates ethylene levels produces ACC deaminases	increase seed germination and dry weight of treated plants. increase the production of reactive oxygen species scavenging enzymes increase leaf chlorophyll and relative water content	Saikia et al. [183]
*Bacillus* spp.	increase relative water content decrease leaf water loss increase sugar contents	increase proline and free amino acids decrease electrolyte leakage increase plant biomass	Vardharajula et al. [190]
*Rhizobia* spp.	produce metabolites like proline and trehalose induce enzymes, e.g., SOD and CAT that can detoxify reactive oxygen species	production of cytokinin, ACC deaminase, abscisic acid, trehalose secretion of volatile organic compounds and exopolysaccharides	Forni et al. [191]
*Bacillus pumilu*	induces tolerance to drought increases the activity of enzymes such as CAT	improve leaf number, tuber size and tuber yield improve plant growth	Gururani et al. [192]

## Data Availability

Data is contained within the article.
